# Effectiveness and safety of escitalopram treatment personalized based on therapeutic drug monitoring of drug plasma concentration: a prospective cohort study

**DOI:** 10.1038/s41598-025-18517-6

**Published:** 2025-09-12

**Authors:** Petar G. Vuković, Aleksandra Jeremić, Milica Vezmar, Filip Milosavljević, Zorana Pavlović, Danilo Pešić, Jelena Drakulić Đorđević, Bojana Pejušković, Bojan Marković, Magnus Ingelman-Sundberg, Čedo D. Miljević, Nađa P. Marić, Marin M. Jukić

**Affiliations:** 1 Department of Physiology, Faculty of Pharmacy, University of Belgrade, Vojvode Stepe 450, Belgrade, 11221 Serbia; 2 Institute of Mental Health, Belgrade, Serbia; 3 Technical University of Munich, Munich, Germany; 4 Department of Psychiatry, Faculty of Medicine, University of Belgrade, Belgrade, Serbia; 5 Clinic for Psychiatry, University Clinical Center of Serbia, Belgrade, Serbia; 6 Department of Pharmaceutical Chemistry, Faculty of Pharmacy, University of Belgrade, Belgrade, Serbia; 7 Pharmacogenetics section, Department of Physiology and Pharmacology, Karolinska Institute, Solnavägen 9, Biomedicum B5, 17165 Stockholm, Sweden

**Keywords:** Escitalopram, Therapeutic drug monitoring, Dose personalization, Depression, Clinical effectiveness, Depression, Clinical pharmacology, Pharmacokinetics, Therapeutics

## Abstract

This is the first prospective study aiming to quantify the effectiveness and safety of escitalopram monotherapy initiation where therapeutic drug monitoring (TDM) was used to achieve the therapeutic reference range (TRR) of plasma concentration. PsyCise-E (NCT05210140) was a hospital-based study conducted in Belgrade, Serbia, involving 92 outpatients with a baseline Hamilton Rating Scale for Depression (HAM-D) score higher than 13. The primary endpoint was the relative reduction in HAM-D score from baseline to week eight, with dose personalization based on TDM four weeks after treatment initiation. Patients were categorized into groups: (1) unadjusted (they achieved TRR at 10 mg/day), (2) adjusted (their dose was adjusted to achieve TRR) and (3) inadequate (they did not reach TRR). Safety was assessed by the occurrence of adverse drug reactions (ADRs) and QTc interval prolongation. Most patients required a dose escalation beyond 10 mg/day (71/92), and most patients achieved TRR after eight weeks (79/92). The 55% (95% CI: 47–64) reduction in HAM-D scores did not correlate with escitalopram plasma concentrations and did not differ between groups; however, response and remission rates were significantly higher in patients who achieved TRR by week four. The incidence of ADRs (47/92) increased by 3.2% (0.1–6.3) per ng/ml escitalopram, with no significant differences between the groups. QTc prolongation of 5.5 ms (1.8–9.3) did not correlate with plasma concentration and did not differ between groups. While TDM-guided dosing likely only marginally improved escitalopram effectiveness, it increased treatment safety as TDM-guided dose escalation did not lead to ADRs or QTc prolongation.

## Introduction

Escitalopram is a frequently prescribed antidepressant^1,2^ with one of the most favorable effectiveness and safety profiles among the 21 most commonly prescribed antidepressants^3^. In the acute treatment of depressed patients, treatment with escitalopram at a fixed dose of 10 mg/day, a fixed dose of 20 mg/day and a flexible dose of 10–20 mg/day was consistently superior to placebo after eight weeks in three randomized controlled trials (RCT)^4–6^. The effect size was about 3.5 points change in the Montgomery and Åsberg Depression Rating Scale (MADRS), with no significant difference in treatment effectiveness between 10 mg/day and 20 mg/day escitalopram^4^. Common adverse drug reactions (ADRs) which are highly dose dependent^2,4,5^ include: insomnia, ejaculatory dysfunction, nausea, increased sweating, fatigue, somnolence, decreased libido, and anorgasmia^2,4–6^. Moreover, a randomized cross-over study with escalating multiple dosing in healthy volunteers has shown that escitalopram prolongs the QTc interval in a dose-dependent manner^7^, which has prompted regulatory authorities to limit the dose of escitalopram to 10 mg/day in patients over 65 years of age and in patients at increased risk of cardiac arrhythmias^8,9^.

Therapeutic drug monitoring (TDM) refers to the quantification and interpretation of drug concentrations in the blood in order to personalize dosing and optimize pharmacotherapy. The consensus guidelines define the recommended therapeutic reference range (TRR) for escitalopram blood concentration between 15 and 80 ng/ml and provide a level two recommendation for the use of TDM^10^; this practically means that TDM is recommended for titrating the dose towards the TRR, for specific indications, or for problem solving. However, the relationship between escitalopram blood concentration, treatment effectiveness and tolerability remains unclear^11^. In particular, a prospective cohort study found an association between escitalopram serum concentration and antidepressant response in 70 depressed patients^12^ and estimated that the threshold concentration required for treatment response is 20 ng/ml, whereas retrospective studies found no such association^13–17^. When patients were categorized into groups based on treatment response, two retrospective studies observed higher escitalopram blood concentrations in responders compared to non-responders^18^ and treatment failure patients^19^. Both studies concluded that 15 ng/ml of escitalopram on average is required for an optimal response to treatment. However, a much larger retrospective study was unable to replicate this observation and found no significant difference in escitalopram plasma concentrations between clinical responders and non-responders^20^. Regarding safety, one retrospective study found a positive association between escitalopram blood concentration and the occurrence of dry mouth^21^, while none of the other retrospective studies conducted to date found a significant association between escitalopram blood concentration and treatment safety parameters^13,14,18,19,21–23^ despite the high dose-dependence of ADRs^2,4,5,7^. However, it is noteworthy that most of the previous reports were retrospective *post-hoc* analyzes of data from very heterogeneous patient cohorts with varying degrees of data representativeness. To our knowledge, no study has prospectively investigated the effectiveness and safety of escitalopram treatment initiation based on TDM-guided dose titration with the aim of achieving the currently recommended TRR. Therefore, this prospective cohort study was designed to resolve the uncertainty stemming from inconsistent and predominantly retrospective findings on the role of TDM in guiding escitalopram treatment.

Accordingly, the aim of this study was to quantify the effectiveness and safety of TDM-guided initiation of escitalopram treatment in a cohort of depressed outpatients and to compare it with the results of previously published fixed-dose studies. For the efficacy comparison, the results of the dose-response meta-analysis by Furukawa et al.^24^ were used, while for the safety comparison, the pharmacovigilance studies presented on the FDA package insert^2^ and supporting data^7^ were used.

## Methods

**Fig. 1:**
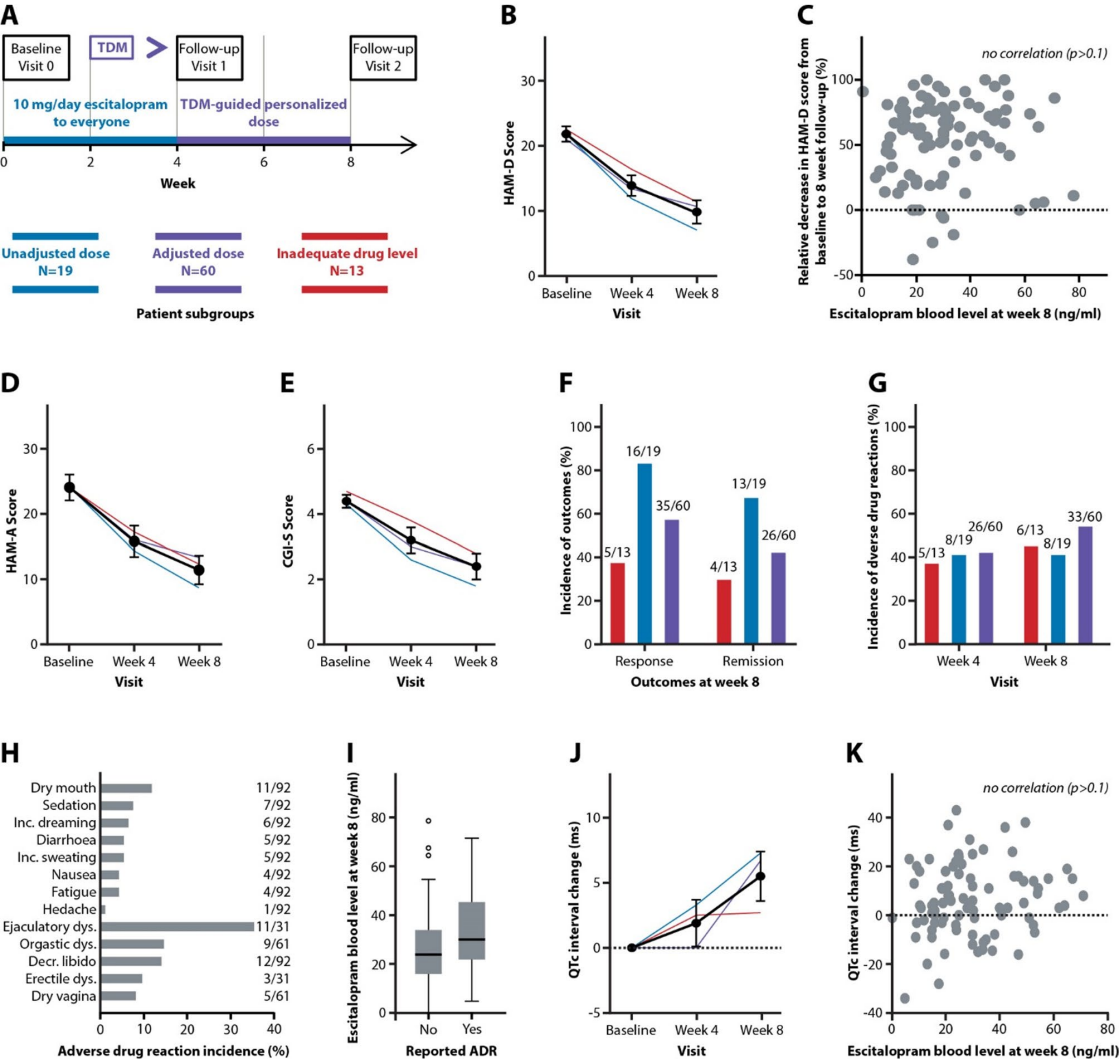
Effectiveness and safety of escitalopram treatment guided by dose personalization based on quantification of drug plasma concentration. (**a**) Schematic representation of study protocol; patients who achieved and maintained TRR with 10 mg/day of escitalopram throughout the trial were labeled as unadjusted dose group; patients who required dose adjustment at week 4 to achieve TRR were labeled as adjusted dose group; and patients who failed to achieve or maintain TRR at week 8 even after dose personalization were labeled as inadequate drug level group. (**b**) Hamilton Rating Scale for Depression (HAM-D) score decreased by 55% (95% CI: 47–64%, *p* < 0.001) from baseline to week 8, without difference in HAM-D score reduction between groups (*p* > 0.1). (**c**) The relative change in HAM-D score from baseline did not correlate with escitalopram plasma concentration (*p* > 0.1) at week 8. (**d**) Hamilton Rating Scale for Anxiety (HAM-A) score decreased by 52% (95% CI: 43–62%, *p* < 0.001) from baseline to week 8, without difference in HAM-A score reduction between groups (*p* > 0.1). (**e**) Clinical Global Impression of Severity (CGI-S) score decreased by 48% (95% CI: 39–54%, *p* < 0.001) from baseline to week 8, without difference in CGI-S score reduction between groups (*p* > 0.1). (**f**) Of 92 patients, 56 responded to treatment and 43 achieved remission; more responders (*p* = 0.0074) and remitters (*p* = 0.036) belonged to the unadjusted dose group compared to inadequate drug level group. (**g**) After week 4 and week 8 of escitalopram treatment, 39 and 47 out of 92 patients reported adverse drug reactions, respectively, without difference between groups (*p* > 0.1). (**h**) Frequency distribution of reported adverse drug reactions at week 8 is depicted from the most to the least frequent. (**i**) Escitalopram plasma concentration increased the probability for the occurrence of ADRs after week 8 by 3.2% (95% CI: 0.1–6.3%, *p* = 0.041) (**j**) QTc interval from baseline to week 8 was prolonged by 5.5 ms (95% CI: 1.8–9.3 ms, *p* = 0.0041), without differences in QTc interval prolongation (*p* > 0.1) between groups. In addition, (**k**) the QTc interval prolongation was not correlated with escitalopram plasma concentration (*p* > 0.1). Results are presented as bar charts, scatter plots and line graphs with annotated estimated marginal means ± 95% CI.

The outpatient recruitment and follow-up were conducted between August 2020 and September 2023 at the Institute of Mental Health in Belgrade, Serbia. The procedures performed during the study protocol implementation are described in detail in the study protocol NCT05210140 (https://www.clinicaltrials.gov/study/NCT05210140) and schematized in Fig. 1A. The study protocol was approved by the Hospital Ethics Committee (approval number: 2083/1). All procedures involving human participants were conducted in accordance with the ethical standards of the institutional and national research committees and with the 1964 Helsinki declaration and its later amendments. Informed consent was obtained from all participants included in the study. The study report was written following the STROBE guidelines (https://www.strobe-statement.org/).

### Study participants

The study inclusion criteria were: (1) age between 15 and 65 years, (2) a score of 14 or more on the Hamilton Rating Scale for Depression – 21 items (HAM-D)^25^, (3) initiation of escitalopram therapy, and (4) signed informed consent from the patient (or legal guardian in case of minors). The exclusion criteria were: (1) severe liver dysfunction (abnormal AST/ALT ratio); (2) severe renal dysfunction (abnormal creatinine clearance); (3) dementia; (4) psychotic disorder; (5) perceived high risk of suicide; (6) drug dependence; or (7) treatment with strong CYP2C19 enzyme inhibitors omeprazole, lansoprazole, pantoprazole, rabeprazole, cimetidine, moclobemide, fluoxetine, fluvoxamine, isoniazid or chloramphenicol. The criteria for study discontinuation were: (1) occurrence of intolerable side effects, (2) patient’s decision to no longer participate in the study, (3) escitalopram levels below 5 ng/ml in the second week of follow-up, indicating poor treatment adherence, or (4) study protocol violation.

### Exposure and measurements

Sociodemographic data included sex and age; cardiac parameters included blood pressure, heart rate, and QTc interval duration based on electrocardiograms; and psychometric data included the HAM-D score, the Hamilton Rating Scale for Anxiety (HAM-A) score^26^ and the Clinical Global Impression of Severity Scale (CGI-S)^27^ score. At follow-up visits, patients were also assessed using the Clinical Global Impression of Improvement Scale (CGI-I)^27^ and the UKU side effect rating scale^28^. Psychometric data at baseline, at four-week follow-up, and at eight-week follow-up visits were collected by a trained rater who remained blinded to both dose and plasma concentrations after baseline visit. Two weeks after starting treatment with 10 mg/day escitalopram, patients’ escitalopram plasma concentrations were measured using a previously validated method based on chromatography and mass spectrometry^29^. After four weeks of treatment with the recommended dose of 10 mg/day escitalopram, dosing was personalized based on TDM results with the aim to ensure that the recommended TRR (15–80 ng/ml) is achieved. Patients continued on 10 mg/day if plasma concentrations were between 25 and 50 ng/ml. If concentrations fell outside this range, doses were adjusted to 5 mg/day (for > 50 ng/ml), 15 mg/day (for 15–25 ng/ml), or 20 mg/day (for < 15 ng/ml). The 25–50 ng/mL interval was pre-specified in the study protocol as a “no adjustment” range, guided by the TRR for escitalopram^10^ and the FDA bioequivalence criterion of 80–125% of the target exposure^30^. This principle was applied to account for expected day-to-day variability in plasma concentrations due to differences in absorption, timing of drug intake before sampling, or occasional missed doses. The lower bound of 25 ng/mL ensures that concentrations remain above the TRR lower limit of 15 ng/mL, while the upper bound of 50 ng/mL ensures concentrations remain well below the TRR upper limit of 80 ng/mL in case of substantial daily fluctuations in drug exposure. Clinicians were informed about plasma levels in relation to the TRR, and dose adjustment guidelines were provided to assist clinical decision-making. At the end of the study in the eighth week, additional TDM was performed to assess whether the escitalopram plasma concentration was within the TRR after the dose adjustment (Fig. 1A).

### Outcomes

The primary endpoint for treatment effectiveness was the reduction in the severity of depressive symptoms, measured as a relative decrease in the HAM-D score from baseline to the eighth week of follow-up. Secondary outcomes included reduction in anxiety symptom severity, measured as a relative decrease in HAM-A scores from baseline to week eight, and changes in global symptom severity and improvement. Global symptom severity was measured by the relative decline in CGI-S scores from baseline to week eight, while global symptom improvement was measured by a CGI-I score of less than four at week eight, indicating at least minimal improvement from baseline. In addition, response rate was measured as the proportion of patients in whom the HAM-D score had decreased by 50% or more from baseline to eight weeks, and remission rate was calculated as the proportion of patients who had a HAM-D score of less than eight at week eight. Safety of treatment was assessed by the proportion of patients experiencing escitalopram-induced ADRs at weeks four and eight, as assessed by the UKU scale, and by calculating the change in QTc interval from baseline to week eight using electrocardiograms. For between-group statistical comparison, the study cohort was divided into three groups: (1) the unadjusted dose group, consisting of patients who achieved and maintained TRR throughout the study with a 10 mg/day dose of escitalopram; (2) the adjusted dose group, consisting of patients who required a dose adjustment at week four to achieve TRR; and (3) the inadequate dose/drug concentration group, consisting of patients who did not achieve or maintain TRR after eight weeks of treatment.

### Statistical data analysis

The statistical analysis was carried out using IBM SPSS 20, while the diagrams were created using GraphPad Prism 8.0.1 software. All quantitative data, such as the decrease in HAM-D score, were analyzed using a two-way mixed analysis of variance (ANOVA), with visit as a within-subjects factor and subgroup as a between-subjects factor. LSD *post-hoc* tests were then performed to assess the statistical significance of differences between groups. All categorical data, including the proportion of patients with ADRs, were analyzed with the chi-square test to assess differences between groups or with binary logistic regression using escitalopram plasma level at week eight, sex, age, and HAM-D baseline score as independent variables. The Person test and Spearman test were used for correlation analyzes for normally and non-normally distributed data, respectively. The p-values below 0.05 were interpreted as a statistically significant difference between the compared groups.

### Comparison of study outcomes with previously published randomized clinical trials

To estimate the influence of the *placebo* effect on the main outcome of the study and to compare it with the results from previously published *placebo*-controlled, randomized clinical trials of fixed doses of escitalopram, the studies selected from the meta-analysis by Furukawa et al.^24^ were reanalyzed. The relative reduction in symptom severity for the placebo, 10 mg/day escitalopram, and 20 mg/day escitalopram arms, measured as the percentage change in HAM-D or MADRS scores from baseline to week eight, was meta-analyzed and compared with the primary outcome of the present study. To estimate the relative reduction in symptom severity for each RCT, the Taylor expansion method^31^ was used to divide the absolute change in symptom severity at eight weeks and the baseline symptom severity score. The calculated relative reductions in symptom severity and their respective standard errors for each trial were then entered into RevMan 5.4 software (Cochrane Collaboration, Foster City, USA) and analyzed using the generic inverse variance option and random effect to obtain grand means for each arm. The grand means of the meta-analysis were then plotted together with the results of the present study and compared by visual inspection. To compare the results regarding the safety of escitalopram treatment between this and previous studies that focused on fixed doses of escitalopram^2,7^ the incidence of distinct ADRs and QTc interval prolongation were compared by visual inspection.

## Results

**Fig. 2:**
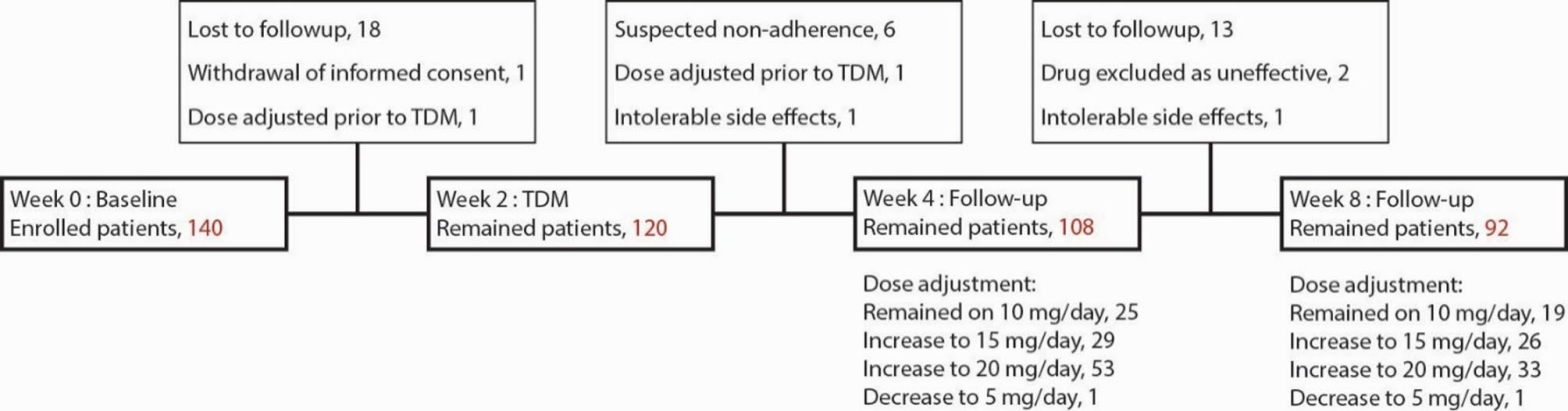
Study flowchart.

Of the 140 patients enrolled, 92 completed the study protocol (Fig. 1A), while 6 participants (4.3%) were excluded due to plasma concentrations below 5 ng/mL measured at week 2 (Fig. 2). The details on cohort status at each phase and reasons for non-participation are presented on the study flowchart (Fig. 2). The mean age of the cohort was 33 years, the mean baseline HAM-D score was 21, and 61/92 participants were women. After eight weeks, 19 patients maintained TRR throughout the study at the escitalopram dose of 10 mg/day, 13 patients did not achieve or maintain TRR, and 60 patients required a dose adjustment; including 26 patients receiving 15 mg/day, 33 patients receiving 20 mg/day and one patient receiving 5 mg/day between week four and week eight. No significant differences in age, gender, HAM-D score or HAM-A score at baseline were observed between the study groups. The baseline data for the entire cohort and individual groups are shown in Table 1.

**Table 1:** Baseline characteristics of the cohort.

Baseline characteristics	Entire cohort	Inadequate drug level	Unadjusted dose	Adjusted dose	*P*-value
Age (years)	33.0 ± 2.5	29.8 ± 7.6	37.4 ± 4.9	32.3 ± 3.3	> 0.1
Number (Female)	92 (61)	13 (9)	19 (13)	60 (39)	> 0.1
HAM-D	21.4 ± 0.9	22.5 ± 3.2	21.8 ± 2.2	21.0 ± 1.2	> 0.1
HAM-A	24.1 ± 1.5	24.1 ± 4.7	24.2 ± 3.1	24.1 ± 2.1	> 0.1

Entire cohort = all patients who completed the eight-week follow-up period and were included in the final analysis. HAM-D = Hamilton Rating Scale for Depression. HAM-A = Hamilton Rating Scale for Anxiety. Data are presented as mean ± 95%CI for age, HAM-D, HAM-A;.

### Effectiveness and safety of TDM-guided Escitalopram treatment

Of the 92 patients who completed the study, 71 required a dose escalation beyond 10 mg/day at week four to achieve the recommended TRR, and 79 were within the TRR at week eight (Fig. 1A). The effectiveness of TDM-guided escitalopram treatment was measured by changes in HAM-D, HAM-A and CGI-S scores from baseline to eight-week follow-up and by achieving a CGI-I score of less than four at week eight. At week eight, the HAM-D score decreased by 55% from baseline (Fig. 1B), with no correlation observed between the reduction and measured escitalopram plasma concentrations (Fig. 1C). Significant improvement was also measured as a 52% and 48% reduction in HAM-A (Fig. 1D) and CGI-S scores (Fig. 1E), respectively, from baseline to week eight, along with a CGI-I score that was significantly below the 4-point threshold at week eight. No statistically significant differences in symptom improvement were observed among patients in the unadjusted dose group, the adjusted dose group, and the inadequate drug level group, as measured by any of the above outcomes (Fig. 1B, D, E; Table 2). Next, response and remission rates were compared between the study groups. Patients in the unadjusted dose group were significantly more likely to have a response and remission than patients in the inadequate drug level group (Fig. 1F). In conclusion, escitalopram dose adjustment resulted in drug exposure within a TRR in the majority of patients and treatment effectiveness was unrelated to escitalopram plasma concentrations in patients within the TRR.

**Table 2:** Parameters in patients treated with dose personalization based on quantification of Escitalopram plasma levels.

Readout	Timepoint	Inadequate drug level	Unadjusted dose	Adjusted dose	*P*-value
HAM-D	Baseline	22.5 ± 3.2 (100%)	21.8 ± 2.2 (100%)	21.0 ± 1.2 (100%)	< 0.0001
	4 weeks	16.4 ± 3.1 (73 ± 14%)	11.9 ± 3.7 (54 ± 17%)	13.4 ± 1.7 (62 ± 8%)	
	8 weeks	11.4 ± 3.6 (50 ± 16%)	7.1 ± 3.3 (32 ± 15%)	10.6 ± 1.9 (52 ± 9%)	
HAM-A	Baseline	24.1 ± 4.7 (100%)	24.2 ± 3.1 (100%)	24.1 ± 2.1 (100%)	< 0.0001
	4 weeks	17.3 ± 5.2 (71 ± 22%)	14.4 ± 4.9 (58 ± 20%)	16.1 ± 2.4 (67 ± 10%)	
	8 weeks	12.4 ± 4.7 (50 ± 20%)	8.8 ± 4.0 (37 ± 17%)	13.4 ± 2.4 (54 ± 10%)	
CGI-S	Baseline	4.7 ± 0.4	4.3 ± 0.3	4.4 ± 0.2	< 0.0001
	4 weeks	3.8 ± 0.8	2.6 ± 0.7	3.0 ± 0.3	
	8 weeks	2.8 ± 0.9	1.8 ± 0.5	2.4 ± 0.3	
CGI-I	4 weeks	2.5 ± 0.7	1.8 ± 0.6	2.1 ± 0.3	< 0.0001
	8 weeks	1.8 ± 0.6	1.4 ± 0.4	1.9 ± 0.3	
QTc interval (ms)	Baseline	392.6 ± 10.1	402.9 ± 8.3	399.5 ± 4.9	0.0041
	4 weeks	395.1 ± 9.9 (2.5 ± 7.1)	406.2 ± 8.2 (3.2 ± 7.5)	399.5 ± 4.7 (0.0 ± 3.6)	
	8 weeks	395.3 ± 8.9 (2.7 ± 10)	410.2 ± 7.4 (7.2 ± 6.6)	406.2 ± 4.4 (6.7 ± 3.9)	
Heart rate (bpm)	Baseline	75.5 ± 9.6	69.8 ± 4.8	76.2 ± 3.5	0.013
	4 weeks	69.7 ± 7.0 (−5.8 ± 6.4)	69.2 ± 5.2 (−0.7 ± 5.3)	73.4 ± 3.5 (−2.8 ± 3.2)	
	8 weeks	70.5 ± 6.0 (−4.9 ± 7.8)	68.1 ± 5.0 (−1.8 ± 4.9)	72.3 ± 2.8 (−3.9 ± 2.8)	
Mean arterial pressure (mmHg)	Baseline	90.2 ± 4.8	91.9 ± 5.0	89.3 ± 2.4	0.069
	4 weeks	88.4 ± 4.4 (−1.8 ± 3.3)	88.9 ± 5.5 (−3.0 ± 5.1)	89.8 ± 2.2 (0.5 ± 2.3)	
	8 weeks	88.0 ± 5.3 (−2.2 ± 4.6)	87.9 ± 4.4 (−3.9 ± 4.3)	88.9 ± 2.8 (−0.4 ± 2.6)	

HAM-D = Hamilton Rating Scale for Depression. HAM-A = Hamilton Rating Scale for Anxiety. CGI-S = Clinical Global Impression of Severity Scale. CGI-I = Clinical Global Impression of Improvement Scale. Data are presented as means ± 95%CI for HAM-D, HAM-A, CGI-S, CGI-I, QTc interval, heart rate, and mean arterial pressure.

Escitalopram-induced ADRs according to the UKU side effect rating scale occurred in 39 and 47 of 92 patients at four and eight weeks, respectively (Fig. 1G), with no difference between study groups (Fig. 1H). Table 3 presents the frequency of the most commonly reported ADRs across treatment groups, organized by symptom category. Escitalopram plasma concentration at week eight was associated with an increased likelihood of ADRs, which increased by 3.2% per one ng/mL increase in serum concentration (Fig. 1I). In addition, arterial blood pressure remained stable during the eight-week period, while heart rate decreased by 3.5 beats per minute and the QTc interval was prolonged by 5.5 ms in the entire cohort. The reduction in heart rate and prolongation of the QTc interval did not differ significantly between the study groups (Table 2). In addition, QTc interval prolongation at week eight showed no correlation with escitalopram plasma concentration (Fig. 1J, K). No patient exceeded the clinically significant QTc threshold of 450 ms at week 8 (Table 2). In conclusion, despite the correlation between escitalopram plasma concentration and risk of ADRs seen in fixed-dose trials, TDM-guided dose escalation did not result in a significant increase in the incidence of ADRs and QTc prolongation compared to patients who remained at 10 mg/day throughout the study.

**Table 3:** Frequency of the most commonly reported adverse drug reactions (ADRs) by treatment group and symptom category.

Adverse drug reactions (ADRs)		Entire cohort	Inadequate drug level	Unadjusted dose	Adjusted dose
Autonomous nervous system	Dry mouth	11/60	2/13	2/19	7/60
	Increased sweating	5/92	0/13	0/19	5/60
Central nervous system	Sedation	7/92	0/13	0/19	7/60
	Increased dreaming	6/92	0/13	0/19	6/60
	Fatigue	4/92	0/13	0/19	4/60
	Headache	1/92	0/13	0/19	1/60
Gastrointestinal	Diarrhea	5/92	1/13	1/19	3/60
	Nausea	4/92	2/13	0/19	2/60
Sexual dysfunctions	Decreased libido	12/92	3/13	3/19	6/60
	Anorgasmia	9/61	1/9	3/13	5/39
	Dry vagina	5/61	0/9	3/13	2/39
	Ejaculatory dysfunction	11/31	3/4	3/6	5/21
	Erectile dysfunction	3/31	1/4	0/6	2/21

Sexual dysfunction items were evaluated by sex: “Decreased libido” in both sexes; “Anorgasmia” and “Dry vagina” in females (*n* = 61); “Ejaculatory dysfunction” and “Erectile dysfunction” in males (*n* = 31).

### Comparison of effectiveness and safety outcomes with previously published data

**Fig. 3:**
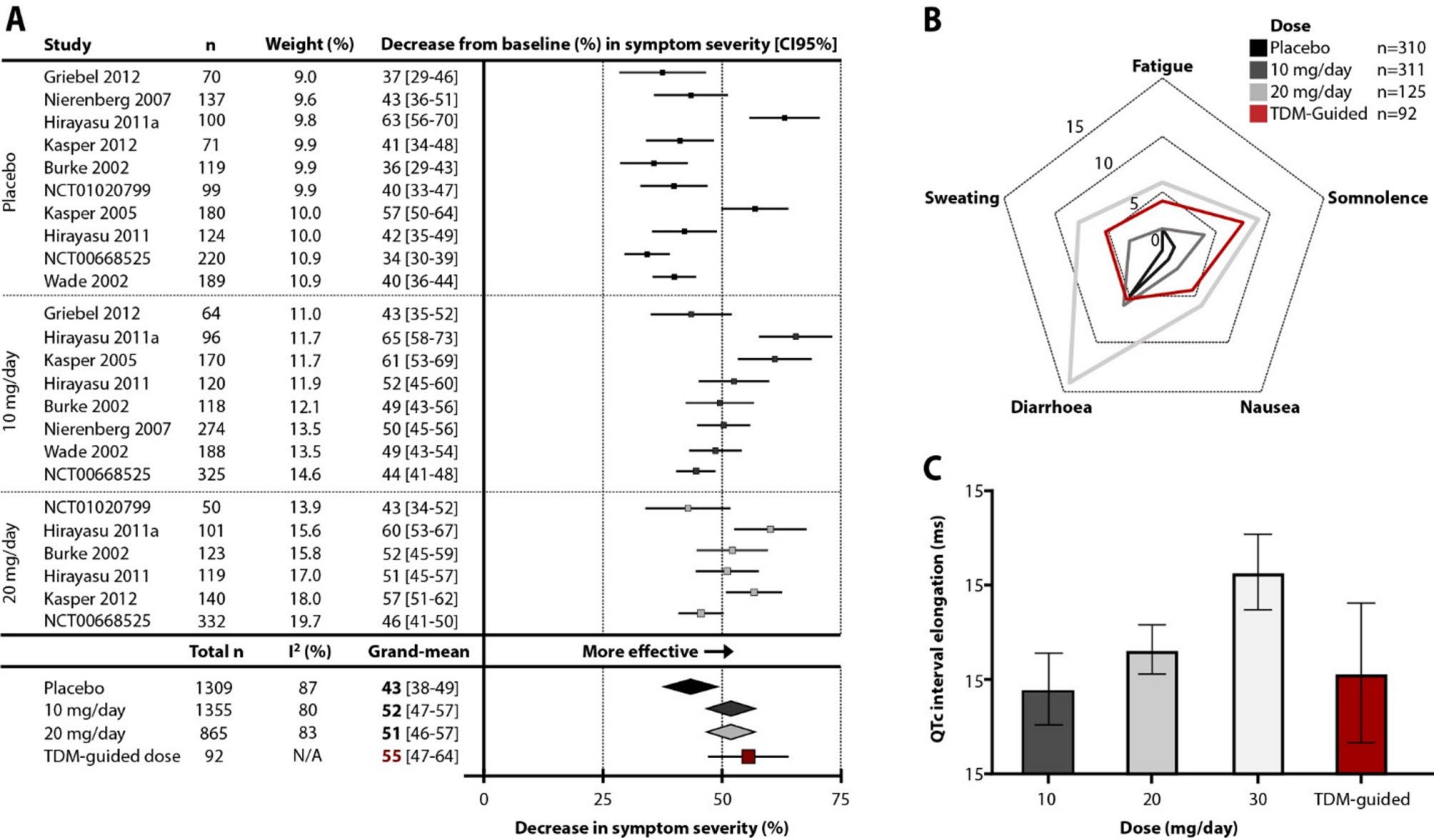
Comparison of the effectiveness and safety of escitalopram treatment in cohorts treated with guided and unguided dosing. (**a**) Meta-analysis comparing the effectiveness of escitalopram treatment between the placebo, 10 mg/day and 20 mg/day arms in fixed-dose randomized controlled trials and the current study using relative symptom reduction on the scale defined as the primary outcome. The placebo arm showed a 43% (95% CI: 38–49%; *N* = 1,309, *p* < 0.001), the 10 mg/day escitalopram arm showed a 52% (95% CI: 47–57%; *N* = 1,355, *p* < 0.001), and the 20 mg/day escitalopram arm a 51% (95% CI: 46–57%; *N* = 865, *p* < 0.001) reduction in symptom severity. (**b**) Comparison of the incidence of the most common adverse drug reactions between the two fixed-dose studies listed on the FDA drug label for escitalopram and the current study is presented indicating that the incidence of adverse drug reactions was between the incidence observed in patients treated with 10 mg/day and 20 mg/day. (**c**) Comparison of the observed change in QTc interval between the current study with TDM guided-dose and the FDA randomized, controlled, cross-over, multiple fixed-dose study. The white and gray bars represent the same cohort (*N* = 113) sequentially exposed to different escitalopram doses, while the red bar represents the current cohort as change from baseline to week 8. The QTc elongation in the present cohort is comparable to the QTc prolongation observed when subjects were treated with 10 mg/day and 20 mg/day. Results are presented as means ± 95% CI.

Of the 12 RCTs in the study by Furukawa et al.^24^, 10 were reanalyzed; The SCT-MD-35 trial was excluded because the escitalopram dose was 4 mg/day^32^, and the NCT00822744 trial was excluded because the effectiveness results for the placebo and escitalopram treatment could not be estimated separately^33^. All RCTs presented data for the placebo, eight RCTs presented data for the 10 mg/day escitalopram, and six RCTs presented data for the 20 mg/day escitalopram. The majority of trial participants were Caucasian. Five studies used the HAM-D scale and five studies used the MADRS scale. Patients treated with 10 mg/day and 20 mg/day escitalopram showed similar reductions in symptom severity of 52% and 51%, respectively, while patients treated with placebo showed a 43% reduction in symptom severity (Fig. 3A).

The incidence of various ADRs in this cohort was compared to those reported in the FDA safety data from the escitalopram package insert for placebo, 10 mg/day and 20 mg/day^2^. The incidence of the most common adverse drug reactions in the present cohort was intermediate between those of the 10 mg/day and 20 mg/day fixed-dose groups, although slightly closer to the 10 mg/day group (Fig. 3B). Next, the observed changes in QTc interval were compared with the results of the FDA randomized controlled cross-over trial of escalating multiple doses in healthy volunteers^7^. The observed mean QTc interval prolongation of 5.5 ms in the TDM-guided cohort studied here was comparable to the mean prolongation of 4.5 ms in the 10 mg/day fixed-dose group and the mean prolongation of 6.6 ms in the 20 mg/day fixed-dose group (Fig. 3C). In summary, the TDM-guided cohort showed a comparable effectiveness and safety profile to the cohorts treated with fixed 10 mg and 20 mg daily doses.

## Discussion

While most patients required a dose escalation to achieve TRR, the majority of patients eventually succeeded to achieve it and responded to escitalopram treatment. While escitalopram blood concentration was not associated with a reduction in symptom severity, response and remission rates were higher in the group that achieved and maintained TRR than in the group that did not achieve TRR. Escitalopram blood concentration was associated with a 3.2% increased incidence of ADRs per ng/ml escitalopram in plasma, but not with QTc interval prolongation. In the TDM-guided settings, dose escalation beyond 10 mg/day, which was required in the majority of patients, did not result in significant safety concerns, in contrast to the studies in which the dose was not guided by TDM and in which ADRs and QTc interval prolongation were more pronounced at higher doses.

The placebo effect resulted in a 43% reduction in symptom severity, while the fixed dose of 10 mg/day, the fixed dose of 20 mg/day and the flexible, TDM-guided escitalopram dose reduced symptom severity by 52%, 51% and 55%, respectively. In the study cohort, no significant difference in symptom severity reduction was observed between the unadjusted dose group, the adjusted dose group, and the inadequate drug level group. Furthermore, the absence of a correlation between escitalopram plasma concentration and symptom reduction is consistent with previous studies^13–17,20^, and suggests that increasing drug exposure beyond the lower limit of the TRR is unlikely to yield additional clinical benefit. Most patients in the present study were within the TRR, and it is likely that escitalopram concentrations above 15 ng/mL do not enhance therapeutic response due to a pharmacodynamic ceiling effect. This interpretation is supported by evidence that serotonin transporter (SERT) occupancy reaches a functional threshold at approximately 8.9 ng/mL (25 nM) of serum escitalopram, a concentration derived from the experimentally determined SERT inhibition constant (Ki = 9.2 nM)^34^, combined with data showing that cerebrospinal fluid concentrations are approximately one-third of serum concentrations^35^, as previously operationalized by Jukić et al.^36^. Moreover, the inherent problem with this and similar studies is a poor signal-to-noise ratio^37^, as more than one-third of participants responded due to the placebo effect and more than one-third belonged to the group of absolute non-responders to escitalopram, regardless of dose. When interindividual variability in relative HAM-D score reduction was circumvented by binary categorization of patients based on symptom reduction, response and remission rates were higher in the unadjusted dose group than in the inadequate drug level group (Fig. 1F). This confirms the previous observation^12^ that achieving a TRR improves the effectiveness of escitalopram treatment. Although the benefit of TDM-guided dosing in terms of effectiveness is likely to be marginal in a naturalistic setting, it may still be important for a subgroup of patients who respond to escitalopram but not placebo; however, the study design and limited sample size do not allow firm conclusions to be drawn on this point. Clarifying the effectiveness of TDM-guided dosing would require a randomized trial with a placebo run-in, as discussed in detail in the Limitations section.

The results also underline the frequent need to increase the escitalopram dose beyond 10 mg/day to achieve TRR. However, such a step is associated with safety concerns, as the incidence of ADRs is dose-dependent^2,4,5,7^ and also blood concentration-dependent, as observed here. Since dosing in this cohort was TDM-guided, patients who were already within the TRR at 10 mg/day were spared the dose escalation that could occur in a naturalistic setting and potentially cause avoidable dose-dependent ADRs. In fact, TDM-guided dose escalation did not lead to an increase in reported ADRs, while in contrast, the ADR incidence in the 20 mg/day fixed-dose group was twice as high as in the 10 mg/day fixed-dose group when the dose was not TDM-guided^2^. Furthermore, TDM-guided dose escalation did not lead to a significant prolongation of the QTc interval. This suggests that clinically meaningful escitalopram-induced QTc prolongation probably occurs mainly at supratherapeutic escitalopram blood levels^7,38^, because at non-TDM-guided dosing, the QTc interval was prolonged by 4.5 and 10.7 ms at doses of 10 and 30 mg/day, respectively^7^. Overall, the TDM-guided treatment approach has the potential to improve the safety of escitalopram treatment, including clinically relevant concerns in patients at higher cardiovascular risk^8,9,38^.

The results discussed suggest that routine use of TDM for escitalopram monotherapy may not be justified with present evidence. Instead, its clinical utility appears to be limited to situations where non-adherence or rapid metabolism is suspected. In this context, TDM plays a dual role: it enables personalized dose titration while also helping to identify patients with likely poor adherence. In the present cohort, 4.3% of patients had escitalopram plasma levels below 5 ng/mL at week 2, strongly suggesting non-adherence. Noteworthy, patients in this cohort knew that TDM will be performed, which implicates significantly higher incidence of non-adherence in naturalistic settings. Importantly, in the absence of TDM, clinicians may misattribute nonresponse to poor adherence or ultrarapid metabolism, leading to unnecessary dose escalation, which is known to compromise treatment safety. If the patient is actually adherent and within the TRR but simply not pharmacodynamically responsive to the drug, such dose increases may result in supratherapeutic exposure and associated ADRs. This highlights the clinical relevance of TDM in protecting patients from avoidable safety concerns while informing better treatment decisions.

Related to cost-effectiveness, which is of relevance for clinical use and reimbursement, the cost of a single TDM measurement is approximately €20–80^39^, while one day in the hospital is 4–16 times more expensive than a single TDM^10^. While to the best of our knowledge, no cost-effectiveness study has been performed specifically for TDM usage in escitalopram monotherapy of depression, TDM can reduce the cost of antidepressant treatment. In particular, treatment cost is reduced by 16% in elderly SSRI users, hospitalization is shortened by 23 days for inpatients treated with citalopram, and treatment failure is prevented by identifying sub-therapeutic concentrations early in the course of treatment^10^. Furthermore, treatment-resistant depression (TRD) is associated with a substantial economic burden, with TRD patients incurring nearly double the annual costs compared to non-TRD patients; in particular, €15,907 vs. €8,335 per patient per year^40^. Numerous TRD cases are in fact pseudo-TRD due to sub-therapeutic drug exposure caused by non-adherence, ultrarapid metabolism, or drug–drug interactions; all these factors can be easily identified by performing TDM^10,41^. Therefore, while it is still unclear whether universal routine TDM for escitalopram is cost-effective, a targeted strategy triggered by specific situations appears economically justified; such situations are: (1) early non-response (2–4 weeks), (2) unexpected adverse effects at low doses, (3) suspected non-adherence, (4) clinically relevant drug–drug interactions, or (5) treatment in special sensitive populations. On the flip-side, situations where TDM is unlikely to add value include (1) stable responders without tolerability issues, (2) late measurements with no planned dose adjustments, or (3) settings where turnaround time is insufficiently fast to improve treatment management. However, unequivocal conclusion about cost-effectiveness of TDM in escitalopram monotherapy of depression for each specific situation would require a dedicated methodological framework and a follow-up cost-benefit analysis.

### Limitations

The main limitations of this study are: (**1**) The limited cohort size of 92 patients meant that certain subgroups were particularly small, most notably the inadequate drug level group, which consisted of 13 patients. This markedly reduces the statistical power to detect subtle but potentially clinically relevant between-group differences, especially for safety outcomes, and increases the likelihood of false-negative findings. Consequently, the results for these subgroups should be interpreted with caution. Future studies with larger and more evenly distributed samples are needed for detection of clinically meaningful effects and quantification of effect sizes. (**2**) The absence of a randomized comparison substantially reduces the ability to draw firm causal inferences about the added value of TDM-guided dosing. Comparisons with historical fixed-dose data provide some context, but cannot fully account for potential confounding variables or time-related biases. Future adequately powered RCTs with TDM-guided and control group treated as usual are warranted to provide unequivocal evidence regarding the clinical utility of TDM-guided escitalopram treatment. (**3**) The high placebo response rate and presence of pharmacodynamic non-responders complicate the interpretation of the marginal group-level benefit observed with TDM-guided dosing. As highlighted earlier^37^, standard antidepressant trials often suffer from poor signal-to-noise ratios due to heterogeneous response patterns, making it difficult to detect robust concentration–response associations. Clinically, this suggests that TDM may not uniformly enhance efficacy across all patients but can offer valuable insights in selected cases, in particular when treatment failure raises questions of adherence, atypical pharmacokinetics, or subtherapeutic exposure. Future studies could benefit from incorporating placebo run-in periods and early response stratification to better isolate pharmacologically responsive subgroups who can benefit from TDM the most, (**4**) The inclusion criteria i.e., age range of 15–65 years, exclusion of patients with severe somatic comorbidities, use of antidepressant monotherapy, and outpatient treatment setting, limit the generalizability of the findings to the following patient categories: preadolescents, older adults, inpatients, individuals with significant medical comorbidities, and those requiring complex antidepressant polypharmacy. To broaden the generalizability and clinical applicability of findings, future studies should include broader and more diverse populations, including older adults, inpatients, and individuals with relevant somatic comorbidities or on complex pharmacotherapy regimens or focus on these distinct categories, (**5**) The pharmacogenetic data, such as *CYP2C19* and *CYP2D6* genotypes were not collected in this study. Variability in escitalopram plasma levels may partly reflect differences in metabolic capacity related to genetic polymorphisms, as well as body weight, renal function, and liver function. Although preemptive genotyping could assist in identifying patients at risk of underexposure or supratherapeutic concentrations, it constitutes a distinct intervention from TDM. Future complementary research should evaluate the potential benefits of integrating preemptive pharmacogenetic profiling with follow-up TDM analysis to improve dose optimization and maximize the utilization of available molecular diagnostic, computational, and treatment monitoring tools.

## Conclusion

The introduction of escitalopram monotherapy by TDM-guided dose titration probably only marginally increases the overall effectiveness of treatment, which may still be relevant for a subgroup of patients who are not *placebo* responders and absolute non-responders. TDM-guided escitalopram treatment improves the safety of escitalopram treatment by preventing unnecessary dose increases beyond the therapeutic reference range, which are associated with the risk of adverse drug reactions and QTc prolongation.

## Data Availability

The datasets generated and/or analyzed during the current study are available from the corresponding author on reasonable request.
